# A Review of GM-CSF Therapy in Sepsis

**DOI:** 10.1097/MD.0000000000002044

**Published:** 2015-12-18

**Authors:** Brittany Mathias, Benjamin E. Szpila, Frederick A. Moore, Philip A. Efron, Lyle L. Moldawer

**Affiliations:** From the Department of Surgery, University of Florida College of Medicine, Gainesville, FL.

## Abstract

Determine what clinical role, if any, GM-CSF may have in the clinical treatment of sepsis in the adult patient.

Advancements in the management of sepsis have led to significant decreases in early mortality; however, sepsis remains a significant source of long-term mortality and disability which places strain on healthcare resources with a substantial growing economic impact. Historically, early multiple organ failure (MOF) and death in patients with severe sepsis was thought to result from an exaggerated proinflammatory response called the systemic inflammatory response syndrome (SIRS).

Numerous prospective randomized controlled trials (PRCTs) tested therapies aimed at decreasing the organ injury associated with an exaggerated inflammatory response. With few exceptions, the results from these PRCTs have been disappointing, and currently no specific therapeutic agent is approved to counteract the early SIRS response in patients with severe sepsis. It has long been recognized that there is a delayed immunosuppressive state that contributes to long-term morbidity. However, recent findings now support a concurrent proinflammatory and anti-inflammatory response present throughout sepsis. Multiple immunomodulating agents have been studied to combat the immunosuppressive phase of sepsis with the goal of decreasing secondary infection, reducing organ dysfunction, decreasing ICU stays, and improving survival. Granulocyte-macrophage colony stimulating factor (GM-CSF), a myelopoietic growth factor currently used in patients with neutropenia secondary to chemotherapy-induced myelosuppression, has been studied as a potential immune-activating agent.

The applicability of GM-CSF as a standard therapy for generalized sepsis is still largely understudied; however, small-scale studies available have demonstrated some improved recovery from infection, decreased hospital length of stay, decreased days requiring mechanical ventilation, and decreased medical costs.

## THE FUTURE OF SEPSIS TREATMENT: IMMUNOMODULATING THERAPY

The incidence of sepsis is expected to rise as the general population ages, and as immune compromising therapies for cancer and autoimmune disease become more prevalent.^1,2^ The septic disease process continues to have a significant economic impact while also straining an already overburdened healthcare system.^3^ Although the *Surviving Sepsis Campaign* has decreased in-hospital mortality of severe sepsis/septic shock from 40% to approximately 30% as compliance has improved, mortality from sepsis is still high, especially long-term, and the search to improve diagnosis and management continues.^2–9^

Earlier recognition and improved management strategies have resulted in an increased rate of survival during the initial acute phase of sepsis. However, this has led to the increased appearance of a new predominant phenotype of chronic critical illness (CCI) which is plagued by increased susceptibility to secondary infections, prolonged ICU stays, long-term cognitive and functional impairment, increased disposition to long-term acute care (LTAC) facilities, and a surprisingly high ongoing post-hospital discharge mortality.^10–26^ We have identified a syndrome that can explain the development of CCI, and have termed it “*a persistent inflammation, immunosuppression, and catabolism syndrome*” or *PICS*.^26^ Immunostimulating adjuvant therapies to combat the immunosuppressive state of late sepsis and improve clinical outcomes have become an area of growing research.^13,24,27–33^ Granulocyte-macrophage colony stimulating factor (GM-CSF), a naturally occurring cytokine that stimulates production and antibacterial function of neutrophils and monocytes, is one of these adjuvants.^34^ It has undoubtedly been one of the most studied immunostimulants in sepsis to date.

GM-CSF has been studied across institutions and age groups with varying indications and dosage. The current body of published research has shown some benefit when examining clinical benchmarks; however, there is a consistent lack of 28-day survival benefit in the adult population.^31,35–37^ This disconnect between improved clinical outcomes and lack of short-term survival benefits is not surprising given the emerging phenotype of PICS which is characterized by long-term disability and indolent death. The effects of GM-CSF therapy on long-term outcomes have yet to be evaluated.

## LATE SEPSIS AND IMMUNOSUPPRESSION

**FIGURE 1:**
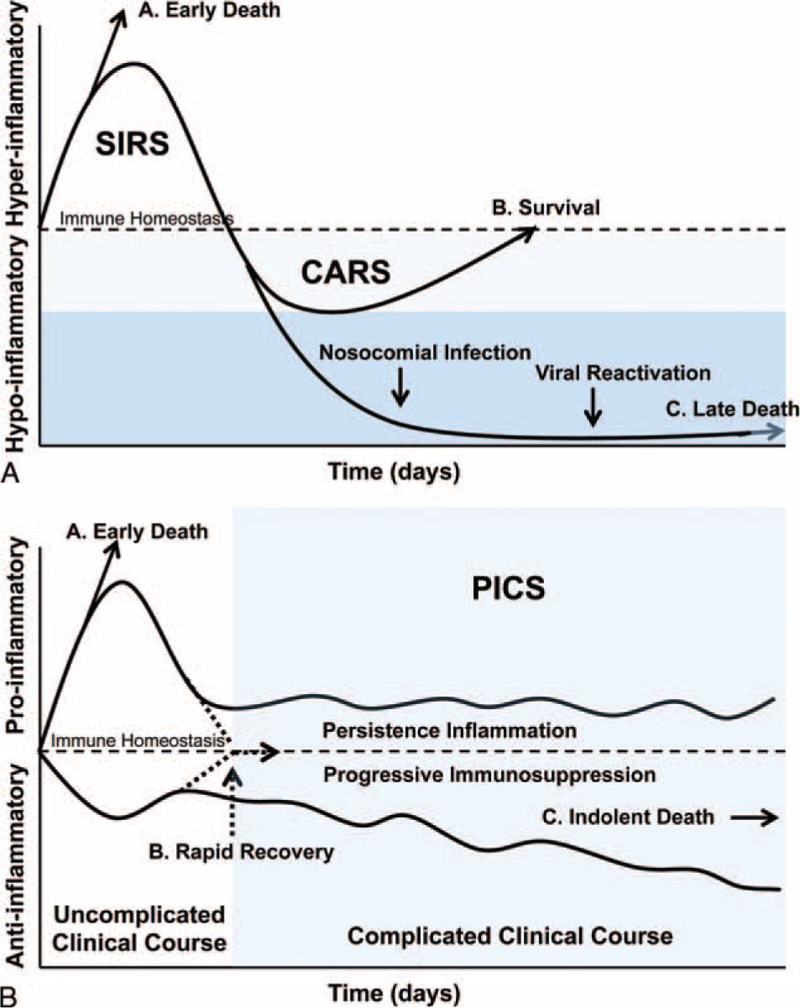
a, SIRS/CARS model of the inflammatory response in sepsis (adapted from [40]). This biphasic view is an oversimplification of a fluid dynamic process demonstrated in (b). Here, (A) early deaths from the acute hyperinflammatory phase of sepsis, (B) survival following acute inflammation (SIRS) followed by a counter regulatory hypoinflammatory (CARS) phase that brings the immune system back into homeostasis, and (C) individuals with immunologic impairment that result in late deaths. b, PICS model of the inflammatory response in sepsis (adapted from [26]). A more accurate schematic of the fluid dynamic process of PICS showing (A) early deaths from the acute hyperinflammatory phase of sepsis, (B) survival following return to a homeostatic immune state, and (C) individuals with PICS immunologic impairment that results in protein catabolism, cachexia, secondary infection, and indolent death following protracted chronic illness. Elderly patients with comorbidities are more likely to suffer from prolonged immunologic impairment and proinflammatory therapies like GM-CSF aimed at preventing this chronic state are currently under study.

Although sepsis is known to cause severe alterations in both adaptive and innate immunity,^4,13,37,38^ the clinical model of the immunologic state during sepsis has been evolving over the last few decades. The once established model of unbridled hyperinflammation, termed the “systemic inflammatory response syndrome” (SIRS) causing early death^39^ spurred the investigation of anti-inflammatory mediators in an attempt to decrease early mortality.^40^ As early survival improved due to advancements made in earlier sepsis recognition and improved critical care management, the emergence of a later immunosuppressive state that leaves the septic patient at risk of secondary infection became apparent.^7,41^ The SIRS model was amended to include a later immunosuppressive state^7,10,11,14,15,19,31,42^ or “compensatory anti-inflammatory response syndrome” (CARS)^43^ which again became the target for immune modulating therapies; however, the focus proinflammatory mediators (Fig. 1a).^13,24,27–32^ Despite extensive preclinical research, no promising immune modulating therapies have been successfully employed.^44^ To date, the implementation and investigation of GM-CSF has been based largely off of a SIRS/CARS model in an effort to combat the CARS phase of immune dysfunction.

As research into the immune state of sepsis continues, our understanding of the course of sepsis has evolved and the biphasic SIRS/CARS model has become less valuable. Surprisingly, this late period of immunosuppression, previously identified as CARS, occurs throughout the course of septic illness and is, surprisingly, associated with low grade, chronic inflammation, and an increase in inflammatory mediators, such as C-reactive protein (CRP), IL-6, IL-1ra, and sTNFR.^43,45^ In addition, alterations in the leukocyte profile with the release of large numbers of immature myeloid cells are also consistent with a simultaneous chronic inflammatory state.^46^ The etiology of both this chronic inflammation and immunosuppression is not known. There are theories that it could be due to exogenous sources such as endotoxin or even immunosuppressive pharmacologic therapies. Others postulate that it is secondary to endogenous molecules such as IL-10,^16,24,37,47,48^ corticosteroids,^23,24,29^ or catecholamines.^36^ As growing evidence mounts that inflammatory and immunosuppressive processes are a fluid ongoing disease process that occurs simultaneously^49,50^ the SIRS/CARS model has been replaced with PICS (Fig. 1b).^26^ The key adaptive immune features that once typified CARS but are now understood to be part of the larger PICS process are immune cell metabolic failure, decreased T-cell numbers, lymphocyte dysfunction and increased apoptosis, increased T cell suppressor function, reduced T-cell repertoire, significant shifts in cytokine polarization toward humoral and T_H2_ cytokines, decreased human, membrane-associated human leukocyte antigen receptors (mHLA-DR), and epigenetic modifications secondary to the cell microenvironment.^4,13,37,48,51–56^ Despite a chronic inflammatory state, innate immune functions are also affected, including impaired phagocytosis, ex vivo production of inflammatory cytokines, cell surface expression of check-point regulators and immunosuppressive molecules, and the appearance of immature myeloid cells with inflammatory and immunosuppressive phenotypes.^57,58^

The magnitude and duration of this immune suppression has been shown to be associated with dramatic declines in clinical outcomes. Patients with immunosuppression, as documented by either reduced mHLA-DR expression, reduced absolute lymphocyte counts (ALC), or ex vivo TNFα production in response to endotoxin stimulation, are associated with increased nosocomial infections.^4,41,59,60^ These poor outcomes range from secondary bacterial infection, consisting of ventilator-associated pneumonia (VAP)^61^ and avirulent opportunistic infections,^62,63^ to reactivation of latent herpes virus such as CMV and HSV,^64^ and an increased risk of multiple organ dysfunction syndrome (MODS).^40,49,65,66^ These patients have increased hospital length of stays, increased disposition to long-term care facilities, and increased long-term mortalities.^26,67^

There have been several proposed methods to combat this immunosuppressed state, from immunostimulating adjuvant therapies to extracorporeal removal of immunodepressants. Immunostimulating treatments that have been studied are interferon γ (IFNγ), IL-7, thymosan α1, and GM-CSF.^13,24,27–32^ Although some studies show improved eradication of primary infection, prevention of secondary infection, and decrease latent virus activation, only thymosan α1 has been shown to decrease 28-day mortality.^32^ However, the importance of 28-day mortality is overshadowed by the more predominant state of chronic disability and indolent death. GM-CSF has been an ongoing area of study over the last couple of decades in populations ranging from the neonate to elderly. While it has been shown to improve clinical markers, it has failed to show a consistent short-term survival benefit.^31,35–37^

The advancements made in the early management of the critically ill have increased short-term survival only to unmask the emergence of a new predominant phenotype of chronic illness, PICS. PICS criteria (Table 1) utilize surrogate clinical markers available in most hospital settings; more specific markers are currently under clinical investigation. Although these patients survive to hospital discharge, they are often discharged to LTAC facilities and often experience hospital readmissions and progress to an indolent death. Short-term survival benefit has not been demonstrated in adult GM-CSF studies; however, our evolving understanding of sepsis toward a model consistent with PICS demands an evaluation of more clinically relevant long-term endpoints such as long-term survival, discharge placement, and return to functional life rather than 28-day mortality. The current understanding of sepsis immunology, one that is consistent with PICS, requires simultaneous management of chronic inflammation and adaptive immunosuppression alongside prevention of secondary infection and severe protein catabolism. Prior studies implemented GM-CSF as a treatment for CARS and failed to recognize the concomitant nature of sepsis immunology. Many have focused on administration of GM-CSF when septic patients transition from SIRS to CARS in an effort to target a late onset immunosuppressive state. Surface expression of mHLA-DR on CD14^+^ blood monocytes has been used as a diagnostic marker of the onset of an immunosuppressive state in several studies.^13,18,21,68^ However, the recognition that immunosuppressive processes are present at the onset of sepsis should challenge the timing of GM-CSF implementation in addition to the end-points evaluated.

**Table 1:**
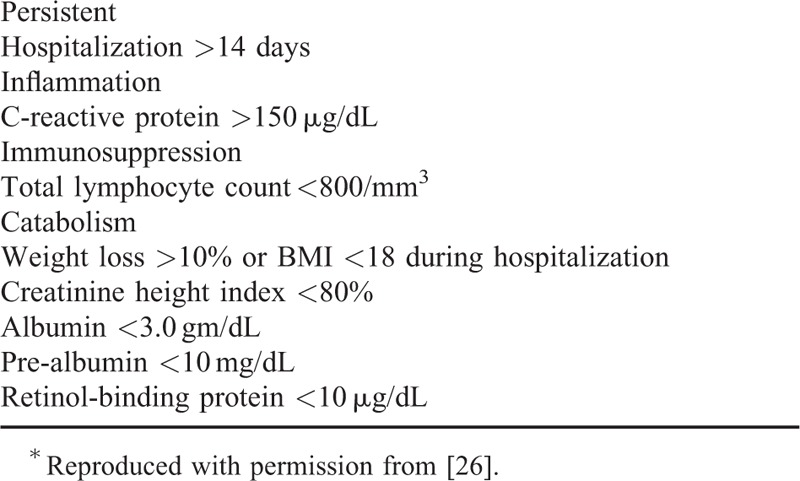
Persistent Inflammation, Immunosuppression, and Catabolism Syndrome (PICS) Criteria^∗^

## MECHANISM OF GM-CSF ACTION OF THE IMMUNE SYSTEM IS EXTENSIVE

**FIGURE 2:**
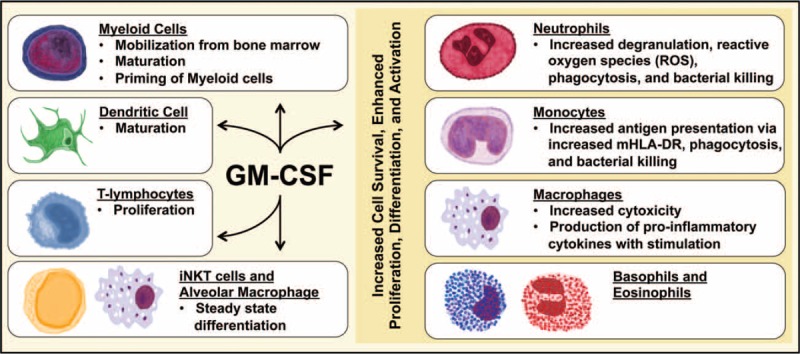
Proinflammatory and steady-state function of GM-CSF. In vitro GM-CSF promotes cell survival, proliferation, differentiation, and activation of neutrophils, monocytes, basophils, and eosinophils. GM-CSF also promotes dendritic cell maturation. In vivo GM-CSF promotes T-cell proliferation. Knock-out (KO) murine models have demonstrated that GM-CSF is involved in steady-state differentiation of invariant natural killer T (*i*NKT) cells and alveolar macrophages. When GM-CSF is administered or released systemically in response to inflammation or infection, it can mimic in vitro effects and promote mobilization of myeloid populations and their precursors into the blood. Full activation of macrophage function requires exposure to both GM-CSF and an additional stimulus such as endotoxin, IL-1, or TNF.

The cytokine GM-CSF is a 23-kD heterodimer first defined by its in vitro ability to stimulate mature myeloid cell expansion, specifically granulocytic and macrophage colonies, from bone-marrow precursor cells.^69^ Later in vitro models demonstrated the effects of GM-CSF on mature myeloid cell populations, and it became clear that its role was more complex than that of simply a hematopoietic-cell growth factor. Its beta-subunit in humans is shared with IL-3 and IL-5, and activation of GM-CSF stimulates at least 3 pathways (JAK-STAT, MAPK, PI3K).^70^ The effects of GM-CSF on monocytes, macrophages, neutrophils, eosinophils, and basophils are polyfunctional and range from increased cell survival to enhanced proliferation, differentiation, and activation (Fig. 2).^70,71^ Interestingly, it has been shown to have a dual nature that is predominantly proinflammatory, but also anti-inflammatory in some instances.

Since its discovery, GM-CSF has been extensively studied and its effects are wide-ranging. The role that it plays as a baseline hematopoietic factor is unclear. In healthy adult populations circulating levels of GM-CSF are inconsistent and often found at very low levels; endogenous production of GM-CSF usually requires inflammatory stimulation.^34,71^ Systemic administration of GM-CSF mobilizes myeloid populations such as monocytes, neutrophils, and tissue macrophages from the bone marrow, and primes these cells resulting in increased in vitro response (ie, cytokine production and superoxide production) when these cells are stimulated with endotoxin.^34,72,73^ This mobilizing effect has been used clinically in patients with myelosuppression secondary to chemotherapy; it has been shown to shorten duration of granulocytopenia and promote immune cell proliferation and function.^74^ There is additional evidence that endogenous GM-CSF plays a role in emergency myelopoiesis in response to infection in addition to its effects on innate and adaptive immunity.^70^

While it clearly has important systemic effects on bone marrow proliferation and differentiation, it also has profound functional effects on existing myeloid cell populations. Further suggesting a proinflammatory role, GM-CSF increases mHLA-DR antigen receptor molecule expression which promotes antigen presenting cells and serves to boost the adaptive immune system.^68^ These dendritic-like cells and antigen-presenting cells are both increased in number and primed for activation by a second stimulus like endotoxin which then results in secretion of significant levels of inflammatory cytokines (TNFα, IL-6, IL-12p70, IL-23).^72,73^ GM-CSF increases neutrophil and monocyte survival, proliferation, differentiation, phagocytosis, and bacterial killing while also increasing cytotoxicity of macrophages.^70,75^ Specific to neutrophils, GM-GSF has been shown to increase bone marrow production and function of myeloid populations including degranulation, reactive oxygen species (ROS), phagocytosis, and adhesion, while decreasing neutrophil migration from tissues effectively immobilizing them at the site of inflammation.^34,58,62^ Clinically, this would be expected to decrease rates of nosocomial infection and opportunistic secondary infections as well as the resulting morbidity and mortality.

GM-CSF knock-out (KO) mice or GM-CSF overexpressing mice have further served to elucidate the wide-ranging functions of GM-CSF. Murine KO models of GM-CSF demonstrate little evidence of impaired myeloid cell development or defense against diverse pathogens; however, these mice do show impaired pulmonary macrophage clearance of surfactant resulting in a pulmonary alveolar proteinosis.^76^ Later studies again using GM-CSF KO models confirmed impairment of pulmonary macrophages;^43,77^ in vitro, there was impaired phagocytosis and secretion of inflammatory mediators which translated to enhanced susceptibility to pneumonia.^58,77^ Intraperitoneal injection in a murine model and introduction of GM-CSF in human peritoneal dialysate increased the number of peritoneal macrophages.^78^ A relative deficiency of GM-CSF during sepsis could explain the propensity toward secondary infection that is improved with endogenous GM-CSF administration. Additionally, these GM-CSF KO mice also have impaired invariant natural killer T (iNKT) cell differentiation during thymic ontogeny as well as some reduced T-cell responses to antigen.^79,80^

Human studies of GM-CSF therapy in sepsis have also documented increased T-cell lymphocytes;^18^ however, the functional capacity of these cells and the possible clinical implications has not been elucidated. When examining a GM-CSF overexpression murine model that is absent of other disease states, macrophage functions, including inflammatory cytokine production, are upregulated and a lethal myeloproliferative state is induced consisting of macrophage accumulation, tissue damage, progressive weight loss, and premature death.^81,82^ These murine models serve to highlight the potential roles of GM-CSF; however, their applicability to the human diseased state which is a complex interplay of cytokines and cells with a varying amount of GM-CSF is limited.

Although GM-CSF appears to have a systemic role and is systemically elevated in states of inflammation and infection,^70,83,84^ there are several studies that implicate the role of GM-CSF as a predominantly local effector. GM-CSF has been extensively studied in autoimmune disease processes and has been implicated in the pathogenesis of autoimmune diseases such as rheumatoid arthritis (RA) and other inflammatory conditions such as asthma. Increased concentrations of GM-CSF are found at sites of inflammation in autoimmune diseases and GM-CSF depletion has been consistently shown to be beneficial in suppressing several autoimmune disease states.^34^ In RA, GM-CSF in synovial fluid acts as a biomarker for disease severity and responsiveness to therapy.^85^ Paradoxically, GM-CSF administration, rather than suppression, has also been proven to be effective in certain instances. While many of these studies have focused on clinical endpoints, there has also been a great deal of immunologic study.

Further supporting GM-CSF as a proinflammatory agent, there is a link between GM-CSF and proinflammatory cytokines TNFα and IL-1, as well as the IL-23-IL-17 pathway.^86^ GM-CSF action in vitro appears to be limited in the absence of additional stimulus (ie, TNFα, IL-1, LPS), and both TNFα and IL-1 increase GM-CSF production by several cell types.^87–89^ Interestingly, GM-CSF can be produced circulating T-cells suggesting a humoral role, or by resident tissue cell types such as keratinocytes, smooth muscle cells, endothelial cells, epithelial cells, and neurons, suggesting a more paracrine effect.^30,88^ The increased production of GM-CSF by various cell types in response to IL-1 and TNFα and the positive feedback have led some to suggest the presence of a “CSF network” that potentiates a chronic inflammatory state.^90^ The ability of GM-CSF to increase host defenses could result in decreased microorganism load and explain the benefits of administration in sepsis.

In stark contrast to these proinflammatory findings, GM-CSF also has the capacity to function as an anti-inflammatory agent. It has been shown to enhance viral replication and promote viral infection,^91^ prevent atherosclerosis progression,^92^ and have dual function, as both inflammatory and anti-inflammatory, in pulmonary fibrosis.^93,94^ The explanation for these findings is unknown but could be attributed to dose, specific disease states, or other as yet undiscovered immune complex interplay. These far-ranging proinflammatory effects spurred a debate regarding timing of administration using the previous model of SIRS/CARS with an attempt to target the later CARS immunosuppressive state.

Since GM-CSF promotes both the number and function of inflammatory cell populations, there has been concern that early administration, during the hyperinflammatory phase, could exacerbate tissue injury by causing a detrimental proinflammatory cascade of cytokines via macrophage stimulation. Therefore, the timing of GM-CSF administration, specifically after the onset CARS, was thought to be crucial to its efficacy. While one study utilized mHLA-DR as a marker for immunosuppression and initiated therapy at a chosen threshold,^18^ there is no standardized method of administration and timing of administration varies in the published literature. However, the emergence of PICS creates an entirely new ideology from which to approach the timing of GM-CSF administration.

## METHODS

The PubMed database was queried using keywords GM-CSF, sepsis, and sargramostim (pharmaceutical analog of GM-CSF). This yielded articles that included pediatric populations, adult populations, and murine models. Pediatric findings were excluded. Journal articles as well as meta-analyses written in English and dealing specifically with GM-CSF as it pertains to clinical outcomes were included. Institutional review board (IRB) approval was not necessary as this is a review paper.

### Administration of GM-CSF in Septic Murine Models and in Human Studies

GM-CSF has been extensively studied in both murine and human models and across age groups. The primary endpoint of interest in these clinical studies has been 28-day survival. Although these studies have universally failed to show a reduction in short-term mortality, several clinical secondary endpoints have been shown to be significantly improved; however, these endpoints may not be generalizable to the heterogeneous population of septic patients. The disconnect between improved clinical status and lack of 28-day survival benefit has led some to conclude that GM-CSF may be of limited benefit; however, the recognition of PICS begs for the evaluation of long-term end-points.

### Murine Models of Sepsis Show Promising Results With Prophylactic Administration of GM-CSF

The murine model of sepsis has well known benefits and drawbacks, especially when attempting to translate and reproduce findings in humans. Specific to GM-CSF therapy is again the issue of timing of GM-CSF administration. The murine model allows administration that ranges not only from early to late, but also as prophylactic administration.

Implementation of GM-CSF therapy in a prophylactic manor in the human sepsis population presents many clinical challenges. However, prophylaxis in a murine model of sepsis has consistently shown increased host resistance to bacteria as well as a survival benefit.^20,35,52^ The implications and feasibility of implementing this in a human model have not been well studied.

Early studies, concerned with the safety of GM-CSF given its potential to exacerbate the exaggerated early inflammatory state of sepsis, studied the effects of GM-CSF administration in a septic model on vital organ function. One study administered a single dose of GM-CSF 3 hours after the induction of peritoneal sepsis by a cecal ligation and puncture model (CLP), and found increased centrilobular degeneration and necrosis in the liver, decreased leukosequestration in the peritoneal cavity suggesting impaired migration, and earlier death.^95^ An ex vivo study has similarly found that GM-CSF inhibits neutrophil migration across IL-1-activated endothelium; interestingly, they also found that GM-CSF enhances neutrophil migration across unstimulated endothelium.^17^ This could potentially explain the findings from murine models of sepsis where early administration is associated with adverse outcomes. The finding of hepatic damage in GM-CSF treated animals has been observed in other GM-CSF studies in murine models. These findings suggest that systemic GM-CSF administration may be detrimental in those clinical conditions associated with an early exaggerated inflammatory response. This study also raises the question of whether GM-CSF should be administered systemically or whether it is meant to act locally at the site of the primary infection.^95^

A later study found improved survival with GM-CSF therapy in a more complex murine model that included CLP, burn, and transfusion.^51^ Survival benefits were also demonstrated in a murine model of trauma.^30^ These studies are not as generalizable to a sepsis model as they include other confounding clinical factors.

### GM-CSF Adult Clinical Trial

Importantly, no human studies to date have shown significant adverse effects from GM-CSF administration. Due to the wide-ranging effects of GM-CSF, it may produce dramatically different results depending on when it is administered in the time course of illness. The difficulty when comparing clinical trials is the lack of homogeneity regarding dosage, route of administration, pharmacologic GM-CSF subtypes used, patient characteristics, and outcomes measured. The primary outcome most frequently studied has been 28-day all-cause mortality. Most studies were performed with implementation of treatment after the onset of sepsis and with the goal of implementing GM-CSF therapy after the acute inflammatory phase had ended, which is based off of the SIRS/CARS model. Both the unpredictable nature of sepsis in an inpatient population and the frequent presence of sepsis on admission make prophylactic administration of GM-CSF in adults challenging, and the efficacy of this prophylactic administration is lacking in the published literature.

### GM-CSF Treatment Improves Clinical Endpoints But Fails to Show Survival Benefit

The number of study participants in published GM-CSF papers is often very low, a meta-analysis is helpful in evaluating the potential benefits for GM-CSF therapy. Four studies, discussed in greater detail below, were included in a meta-analysis that consisted of 12 placebo-controlled randomized controlled trials (n = 2380) that included GM-CSF (n = 4 RCTs)^22,38,68,96^ as well as another therapy (n = 8 RCTs).^97^ There was no difference in 14-day, 28-day, or in-hospital mortality; however, earlier resolution of infection was significantly increased and there were no significant adverse events. The drawback of this meta-analysis is that GM-CSF was not the sole therapy studied in several of the studies, and was the focus of study in a minority of papers. A major critique of many early studies was the implementation of GM-CSF therapy without documentation that the patient was in a state of immunosuppression. To address this, a multi-institutional trial has utilized mHLA-DR expression on CD14^+^ blood cells as a biomarker for severity of immunosuppression, and subsequently monitored immune recovery with GM-CSF therapy.^18^ This importance of the timing of administration since the recognition of PICS is less clear; due to the concurrent nature of inflammatory and anti-inflammatory processes, one might argue for the implementation of GM-CSF at the onset of disease. While there was again no difference in 28-day mortality, there were significant improvements in alternative clinical end-points as detailed below.

### mHLA-DR Biomarker-Guided Therapy: A Stratification of Patient Need

In an attempt to quantify immune suppression, mHLA-DR expression was studied as a potential biomarker to both diagnose immune suppression and monitor response to therapy.^68^ Lower mHLA-DR expression has been shown to be indicative of poor immune cell function,^14^ increased rates of nosocomial infection,^13^ and has been associated with reduced survival;^12,29,42,48,67,98,99^ however, some controversy remains as some authors did not find this association.^48,100^ One study was not powered to evaluate mortality, which was not significantly different. Their goal was to evaluate mHLA-DR expression as a possible biomarker of sepsis, and to validate previous ex vivo studies showing increased mHLA-DR expression in response to GM-CSF while tracking clinical end-points to 28-days. This was a prospective, randomized, double-blind, placebo-controlled multi-institutional study (n = 38) that evaluated patients in a state of severe sepsis or septic shock with low mHLA-DR expression. These patients were treated for 8 days with GM-CSF therapy and their outcomes were followed for 28 days.

This study found that GM-CSF therapy increased mHLA-DR expression; however, the study unfortunately did not fully assess monocyte function. Increases in neutrophils, monocytes, and T-lymphocytes were documented but again, the function of these cells was not fully assessed. A change in cytokine profile toward inflammatory cytokines (IL-6, TNFα) with a concurrent decrease in anti-inflammatory cytokines (IL-10) in the GM-CSF-treated patient population was observed. Clinically, GM-CSF therapy significantly decreased length of mechanical ventilation, and there was a trend toward decreased ICU and hospital days as well as disease severity score. Of note, the control group had a trend toward more severe disease. No adverse events directly attributed to GM-CSF therapy were reported.^18^ Although this study noted quantitative differences, they did not study the qualitative differences; however, a prior study did examine the effects of GM-CSF on leukocyte function.

### GM-CSF Increases Leukocyte Number and Function and Improves Clinical End-Points

A randomized, un-blinded, placebo-controlled prospective study (n = 40) evaluated GM-CSF efficacy on clinical markers to 28-days, and on leukocyte function in septic patients.^96^ Of note, one-third of the study population was undergoing organ transplant, severe sepsis and septic shock were excluded, and there was no standardization of antibiotic regiment. The GM-CSF group was found to have a significantly higher increase in total leukocyte counts which corresponded to increased rates of infection clearance and clinical improvement. However, there was again no observable difference in mortality. Ex vivo assays demonstrated an increase in inflammatory activation in GM-CSF-treated patients. Specifically, there was an increase in adhesion molecules that aid in transendothelial migration of neutrophils and monocytes. This study additionally confirmed an increase in mHLA-DR expression in the GM-CSF-treated patients.^96^ While the clinical efficacy of GM-CSF appeared limited to the rate of infection clearance, this is a largely underpowered study and more homogenous septic populations have resulted in additional significant improvements in clinical markers.

### GM-CSF Significantly Impacts Clinical End-Points in Selected Patient Populations

To control for the heterogeneity of the septic population, 1 trial limited patient selection to abdominal sepsis. A randomized, double-blinded, placebo-controlled clinical trial (n = 58) studied GM-CSF effects in patients with nontraumatic abdominal sepsis that underwent surgical intervention.^22^ Only clinical markers were measured, leukocyte number and function was not reported. There were multiple significant improvements in clinical end-points. GM-CSF significantly decreased hospital length of stay, median duration of antibiotic therapy, number of infectious complications, and direct medical costs.^22^ While this study has limited applicability to the larger heterogeneous population of septic patients, it further reinforces with the idea that GM-CSF therapy may be beneficial especially in subsets of patients.

### Protective Pulmonary Effects of GM-CSF

An additional study again chose to focus on a specific patient population consisting of patients with severe sepsis and respiratory dysfunction. This study was a randomized, double-blind, placebo-controlled study (n = 18) that showed improved gas exchange which corresponded to decreased alveolar neutrophils.^38^ There was also increased function of circulating neutrophils and pulmonary phagocytes. Again, no survival benefit was found and no adverse events attributable to the drug were documented.

There are, unfortunately, many limitations to the conclusions that can be drawn from the current body of research since most of the studies are small and have been underpowered for clinical outcomes, especially survival. However, multiple studies have demonstrated the benefits of GM-CSF therapy on clinically significant patient end-points, and 1 study on medical costs. Further research is needed to identify those patient populations most likely to benefit from GM-CSF treatment and larger trials in these patient subsets may provide more insight into possible survival benefits. Although no adult trials have documented survival benefit from GM-CSF therapy, there has been a significant survival benefit in neutropenic septic neonates. In the context of PICS immune dysfunction the role of GM-CSF in preventing chronic disease states progressing to indolent death demands to be studied. GM-CSF appears to combat some of the immune processes that are attributed to PICS as detailed earlier and the potential of this agent, possibly in combination with other agents, may be the as-yet undiscovered therapy to prevent and combat PICS.

### GM-CSF Improves Survival in Neutropenic Septic Neonates

The elderly and the very young have been shown to have unique immunologic deficits that predispose them to increased rates of sepsis and increased resultant morbidity and mortality.^101,102^ Due to differences in their baseline immune function, it would follow that they may have differing responses to GM-CSF therapy when compared with the adult population. Although there have not been any studies focusing specifically on the elderly, the clinical impact of GM-CSF in the neonatal population requires a separate review because of the extensive body of published research. This body of research is growing, and like the adult studies there have been promising results in several clinical end-points. Interestingly, there also appears to be a significant impact on survival in a small subset of neonatal neutropenic patients. A Cochrane review published in 2003 evaluated 7 studies that examined administration of GM-CSF after the onset of sepsis and 3 studies that employed GM-CSF as a prophylactic therapy.^103^ While they found no decrease in all-cause mortality, there was significant reduction in mortality in neonates with systemic infection and neutropenia found in a prior meta-analysis.^104^ However, these studies had small numbers and require further validation. Like the adult population, research into the applicability of GM-CSF therapy in subpopulations of septic patients is ongoing.

### Shortcomings of Available Research: GM-CSF Versus G-CSF

Although GM-CSF and granulocyte-colony stimulating factor (G-CSF) have many overlapping downstream effects, they also have functions that are unique. G-CSF has been shown to more heavily impact antimicrobial defense, while GM-CSF has been shown to restimulate antigen-presenting cell function and bolster adaptive immunity.^34^ Studies that either focused on G-CSF or failed to differentiate between the 2 are therefore difficult to interpret. The failure to recognize GM-CSF and G-CSF as distinct compounds is a shortcoming of several available meta-analysis publications.

### Heterogeneity of Trials

Although GM-CSF is FDA-approved for chemotherapy-induced neutropenia, its off-label use for sepsis has resulted in highly variable dosing regimens. This has led to varying routes of administration and dosage among publications; some studies gave continuous infusion while others implemented several days of varying dosages and dosage intervals. The potential differences of implementing GM-CSF systemically versus locally have also not been evaluated; systemic administration appears to effect a mobilization from the bone marrow while local administration likely affects the proinflammatory effects demonstrated in vitro. In contrast to murine models that prophylactically implemented GM-CSF, essentially all of the human adult studies implemented GM-CSF therapy after the onset of disease. The timing for starting GM-CSF therapy varied; some studies started administration at the time of diagnosis, others chose a specific day status-post onset of sepsis (ie, day 5), while others measured biomarkers thought to be indicative of an immunosuppressed state and only administered GM-CSF once the patient was proven to be in an immunosuppressed state. A critique of many of the early papers is the failure to stratify patients according to their immunological states by utilizing biomarkers or direct measures of immune function; however, in the context of PICS this concept needs to be re-examined. Another variable to consider is the lack of standard manufacturing of GM-CSF resulting in pharmacologic subtypes, mostly varying in their glycosylation patterns. Additionally, the variability in patient characteristics alone can be difficult to control for and the inclusion and exclusion criteria varied between studies; some studies specifically evaluated severe sepsis and septic shock while other studies excluded these populations. Patient selection is further obfuscated by comorbid conditions, age, and genetic differences that are in some cases impossible to take into account. These trials often enter patients who will have good outcomes regardless of the intervention. The complexity of these variables could explain some of the observed differences across publications and the lack of cohesive study findings.

### Heterogeneity of Sepsis

Sepsis is a complex disease state with variable etiologies.^105^ Onset of disease can be secondary to infection from a number of organ systems. Infectious agents can vary from bacterial, to fungal or viral. The severity of the disease burden can vary extensively and can be difficult to quantify. Classification of sepsis into subcategories can be difficult. The feasibility of studying only 1 subtype of sepsis can limit enrolment numbers and present a challenge of power to the study. The analysis of certain subtypes of sepsis also limits the applicability of study findings to a broader patient population. The heterogeneity inherent when studying sepsis has likely been a major factor over the last several decades of pharmaceutical research; there are still no Food and Drug Administration approved drugs in the treatment of sepsis.^106^

### Concluding Remarks and Future Perspectives

Sepsis continues to be a significant source of morbidity and mortality despite advancements made over the last few decades. Many of these advancements have been in the recognition and management of the early acute hyperinflammatory sepsis or SIRS; however, these successes have led to the realization of a later stage of sepsis anti-inflammatory CARS. On further study, this biphasic model is an oversimplification of a more complex immune phenomenon of concurrent inflammation and immune suppression that can lead to a state of chronic inflammation with concurrent immunosuppression, termed PICS. This state leaves a patient not only vulnerable to secondary infection but in a state of protein catabolism that eventually leads to indolent death. The advent of CARS resulted in a growing body of research focusing on immunostimulatory agents to bolster the immune system and to improve outcomes without inducing exaggerated inflammatory activities. GM-CSF, a growth factor, with wide-reaching effects on the immune system has been studied in murine, ex vivo, and human models with the hope of improving survival to microbial infections and sepsis. The current body of research has many short-comings, 1 of which is studies that are underpowered to detect survival advantage and that examine only short-term survival. There has been no evidence of short-term survival benefit from GM-CSF therapy; however, select patient populations have demonstrated significantly improved clinical outcomes without deleterious side effects. These clinical improvements include more rapid recovery from infection, decreased hospital length of stay, decreased days requiring mechanical ventilation, and decreased medical costs. The widespread efficacy across all septic patient populations appears to be limited; however, for patients with abdominal sepsis and pneumonia, implementation of GM-CSF may improve short-term clinical outcomes and decrease direct medical costs.

These findings, specific to GM-CSF, bring up several important questions when approaching immune-modulating treatment for sepsis. While GM-CSF does not appear to have a significant impact in all septic patient populations, there does seem to be a subset of patients that may benefit from therapy. For example, Presneill et al suggested that GM-CSF may have a homeostatic role in sepsis-related pulmonary dysfunction. It may be that the heterogeneity of septic foci and severity play a dominant confounding role and that select patient populations need to be defined and studied as largely separate entities. In addition, the heterogeneity of immune dysfunction present in septic patients means that targeting a sole cytokine or implementing a single agent therapy may not be able to significantly impact morbidity and mortality. Defining patient-specific immune deficits, finding diagnostic techniques that can be implemented in clinical practice, and then targeting these defects with a cocktail of therapies may be the only way to significantly impact mortality.

The studies reviewed here were influenced by the SIRS/CARS model and they were implementing GM-CSF therapy in response to a late CARS state. However, PICS with a concurrent state of pro- and anti-inflammatory processes demands a re-evaluation of the timing of administration and pertinent end-points to be evaluated. The predominance of early death or death in the hospital setting from sepsis has been decreasing due to advances in critical care management and is giving way to a chronic disease state that leads to a late indolent death. It is this profound disability and late death that needs to be closely evaluated and targeted. The far-reaching immunologic impact of GM-CSF therapy should be able to combat many of the immunologic derangements that characterize PICS; the potential of GM-CSF to prevent and combat a PICS disease state is promising. A large prospective multi-institutional study with standardized administration of GM-CSF to all sepsis patients should be performed. The lack of significant GM-CSF deleterious effects argues the safety of broad application in early trials. The goals should be to stratify patient and disease states to identify subpopulations most likely to benefit from therapy. The end-points should be clinically relevant to pertinent in-hospital endpoints as well as long-term disability, hospital readmission, return to functional life, and late death.
